# Relationship power and HIV sero-status: an analysis of their relationship among low-income urban Zimbabwean postpartum women

**DOI:** 10.1186/s12889-019-7137-y

**Published:** 2019-06-21

**Authors:** Teurai Rwafa, Simukai Shamu, Nicola Christofides

**Affiliations:** 1 School of Public Health, Faculty of Health Sciences, University of the Witwatersrand, Johannesburg, South Africa; 2 Health Systems Strengthening Division, Foundation for Professional Development, Pretoria, South Africa

**Keywords:** Gender, Relationship power, HIV, Women, Zimbabwe

## Abstract

**Background:**

HIV disproportionately infects women in many regions. Zimbabwe is one of the countries, most heavily affected. Unequal gender power relations between men and women can increase women’s vulnerability to HIV. The aim of this paper was to determine the relationship between gender power and HIV sero-status among postpartum women in Zimbabwe.

**Methodology:**

A cross-sectional survey was conducted among 2042 women aged 15–49 years, attending postnatal-care at six public primary health care clinics in low-income urban communities of Harare in 2011. Women were asked about relationship power factors using an interviewer-administered questionnaire. The questionnaire included adapted WHO multi-country study items, which measure partner violence perpetrated against women. HIV status data were based on rapid HIV diagnostic tests done during earlier antenatal visits. The analysis was restricted to women with known HIV test results (*n* = 1951). Multivariable logistic regression analyses were performed to assess the predictors of HIV and relationship power factors.

**Results:**

HIV prevalence was 15.3% (*n* = 299/1951). Three quarters of the women (76.9%, *n* = 1438/1871) reported some level of relationship control in their current/most recent intimate relationship. HIV positive women reported higher levels of control by the male partner in their intimate relationships. In adjusted models, the study found a significant association between relationship-control by the male partner and women’s HIV status (AOR 1.11, 95% CI 1.01–1.22), and the decision-making dimensions of relationship power. Although there were indications of high male partner control in participants’ intimate relationships, some women still had agency, as they were able to make independent decisions to fall pregnant. These women were less likely to be HIV positive (AOR 0.54, 95% CI 0.29–1.00). Having a partner who ever refused use of a family planning method was associated with increased odds of having a positive HIV status among the postpartum women (AOR 1.88, 95% CI 1.20–2.90).

**Conclusion:**

Unequal gender power relations continue to be a risk factor for heterosexual transmission of HIV. This suggests that prevention efforts have not successfully resulted in gender equality. HIV prevention interventions should address gender power dynamics to help curb the disproportionate HIV burden among women.

## Background

Women and girls are at a higher risk of being infected by HIV across the world [1]. Evidence suggests that gender inequity and intimate partner violence (IPV) may contribute to this risk [2, 3]. Southern Africa harbours the highest prevalence of HIV and AIDS in the world [4, 5]. Being female is associated with higher rates of HIV infection in sub-Saharan Africa [3, 6]. High levels of HIV sero-prevalence among women in Southern Africa reflect deep-rooted social and gender inequities [7, 8]. The term ‘gender’ is used to describe a social construct, or set of norms, which dictate the amount of power, responsibilities, and obligations men and women have in society [9]. While gender dynamics differ across sub-Saharan Africa, there are some commonalities which increase women’s vulnerability [10, 11]. Unequal power relationships between women and men may contribute to women’s risk of HIV transmission [11–15], as they affect sexual decision-making and the ability of women to use condoms [16–19].

Zimbabwe is one of the countries, most heavily affected by HIV [20], with over 70% of patient admission due to HIV-related illnesses [4]. At the end of 2017, 1.3 million people were estimated to be living with HIV [21]. HIV prevalence is 13.8% among 15–49 years [20], compared to a global estimate of 0.8% among the same age group [6, 22]. In Zimbabwe, HIV prevalence rates hugely differ across all its ten administrative provinces; with a minimum of 11.9% and a maximum of 21.5% [20]. In Harare, where the study was conducted, the HIV prevalence is similar to the national figure. Urban areas have a slightly higher HIV prevalence than rural areas (14.3% vs. 13.5%) [20]. Women are disproportionately infected by HIV, with a prevalence of 16.7% compared to 10.5% among men [20]. HIV prevalence among pregnant women (15–24 years) has been estimated to be at 9.8% [23]. This data reflects a very high burden of disease due to HIV in the country. Gender differences in HIV risk can increase vulnerability due to the interplay of biology with the social construction of gender, including the gendered power relationships between women and men, as well as the direct impact of structural gender inequalities [19, 24], among women in Zimbabwe.

Manifestations of unequal power in intimate relationships extend to decision making about reproduction. Examples include men’s fertility control and decision-making about the number and spacing of children [14]. Relationship power in this study was defined according to the Theory of Gender and Power as having power to act, or change, or having power over one’s sexual partner [17]. There are two conceptual dimensions of relationship power in heterosexual relationships: control in intimate relationships and decision-making dominance [15, 17, 25]. In addition to decision-making, unequal power in relationships manifests in terms of rights, income, wealth, healthcare access and opportunities [26].

IPV is an extreme manifestation of unequal power in relationships. IPV not only violates women’s rights, but can increase the risk of HIV transmission [27]. While there is an association between IPV and HIV, especially in Southern Africa [28], causal links are hard to investigate. Some authors hypothesize that the association that has been noted between IPV and HIV may be due to the fact that male perpetrators are more often HIV infected than other men, due to their riskier sexual behaviour. An association between IPV perpetration and risky sexual behaviour has been established in Southern Africa [29]. Other authors suggest that the HIV epidemic has contributed to higher levels of IPV [30–32], for example, a woman disclosing being HIV positive can trigger violence from partner [31–33].

Pregnant and post-partum women are a special group of women. They have more regular contact with health services through accessing mother-and-child programmes. Postnatal care (PNC) provides an opportunity for identifying pregnant women who have experienced controlling behaviours and IPV [34]. Research has shown that unequal gender-power relations between men and women result in male controlling behaviours and IPV which leads to HIV treatment adherence challenges for women and may affect viral suppression [35–37]. In addition, children may be at increased risk of HIV infection [35]. A study in South Africa found that partner violence alters adherence to antiretroviral treatment among women living with both IPV and HIV [35]. A study in Zambia found, similar results among pregnant women eligible to enrol in the prevention of mother-to-child transmission of HIV programme [37]. Partner relationship factors have been identified as a barrier to HIV treatment outcomes among pregnant and postpartum women in sub-Saharan Africa [38].

The high burden of HIV in Zimbabwe that affects more women than men makes it crucial to study the dynamics of relationship power and HIV infection in postpartum women. Young women fail to protect themselves against gender norms that provide men with control over women’s sexuality and decision-making that may fuel HIV infection in Zimbabwe [11, 14]. Understanding relationship power among women, particularly the pregnant and post-partum sub-population may help to influence HIV interventions in Zimbabwe. Therefore, the aim of this paper was to determine the relationship between gendered power as defined by the relationship control score, decision about most recent pregnancy and current/recent partner ever refusing a family planning method, measured against HIV sero-status, among low-income urban Zimbabwean postpartum women.

## Methods

**Fig. 1:**
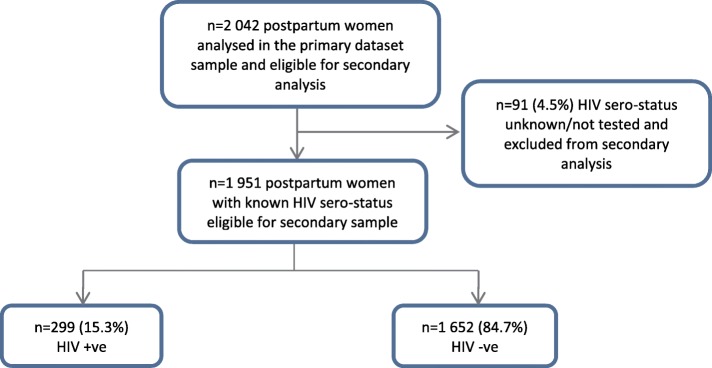
Flow Chart- Inclusion and Exclusion Criteria

A cross-sectional survey was conducted among 2042 postpartum women aged 15–49 years, attending six public low-income urban primary health care (PHC) clinics in Harare at 10 days or 6 weeks post-natal care (PNC) visit in 2011. Sample size was calculated at 2024 participants, based on a similar study conducted in South Africa [39]. The six clinics were purposefully selected according to their history of a working relationship with a teaching programme [34]. All participants queuing for PNC services were offered an opportunity to participate in the study. A detailed methodology of the study has been reported elsewhere [39]. An adapted World Health Organization (WHO) multi-country study questionnaire which measures violence perpetrated against women was used [40]. The questionnaire was pre-tested among 60 post-natal women at a clinic similar to those selected [39]. Data were collected in the local language, with a response rate of 97.1%. The primary dataset was based on the final 2042 women who were interviewed postnatal at either 10 day (56.6%, *n = 1156*) or 6 week PNC visit (43.4%, *n = 886*) [39]. This secondary analysis was restricted to women with known HIV test results (*n = 1*951). A sample is considered statistically significant if missing data is less than 20% [41]. Overall, the sample size was big enough to detect differences in the associations. Figure 1 shows the inclusion and exclusion criteria followed.

### Measures

HIV sero-status was the outcome variable of interest. HIV results were based on HIV rapid tests done on the participants at the clinics during their most recent antenatal care (ANC) visits. HIV status information were collected through matching participants and their ANC records at the clinics, including use of ANC cards which indicated if a woman had been dispensed with neverapine during pregnancy or not, especially for women who had not tested at the selected clinics. During the time of the study, clinics were using rapid test (Determine™) to test for HIV status; positive results were confirmed using a Capillus test. The Western blot test was used to resolve discordant results.

Exposure variables were measures of relationship power defined as relationship control and decision-making power in intimate relationships. Relationship power was defined as having control over oneself or the intimate relationship, and being able to make sexual and reproductive health decisions as measured by the Sexual Relationship Power Scale (SRPS). The SRPS uses two sub-scales: relationship control in intimate relationships and, decision-making. Control in intimate relationships compared women who had no control with women who had some level of control in their relationships [42]. Relationship control was measured using a score created through summing up a six item sub-scale (Cronbach’s alpha = 0.60). Women were asked to respond yes or no to the following questions: does your partner try to keep you from seeing friends, try to restrict contact with your family, insists on your whereabouts always, gets angry if you speak with another man, is often suspicious that you are unfaithful and expect you to ask his permission before seeking health care for yourself. These responses were then added together to create a minimum and maximum score of zero and six respectively. Decision-making was measured using two scenarios; (i) decision to fall pregnant for the most recent pregnancy and, (ii) if male partner ever refused the woman to use any family planning method. Women were asked to answer questions about who made the decision about your most recent pregnancy, and did your current/ most recent partner ever refused a family planning method.

In order to characterise HIV risk, we assessed sexual history and relationship characteristics in terms of age difference with partner, first forced sex, transactional sex, sexually transmitted infection (STI), condom use, and alcohol use. These variables were described as follows: women’s age difference with partner was measured by subtracting the woman’s age from that of her current/most recent male partner. Forced first sex (including rape) before the age of fifteen, was aggregated by adding women who reported that they were forced to have sex and those who reported rape. Transactional sex was measured by asking women if they had ever stayed with a partner longer than they wanted because they were worried they could not afford to provide resources such as shelter, money, gifts, and clothes by themselves. To establish a history of STI infection women were asked to self-report if they were ever treated or diagnosed of an STI during their most recent pregnancy. Ever condom use was measured by asking women if they had ever used a condom with their partner. Alcohol use was measured by asking women how often they drank alcohol in the past 12 months before their recent pregnancy, and how many times they drank alcohol during their most recent pregnancy.

### Statistical analysis

Analyses were restricted to 1951 (95.5%) post-natal women whose HIV test results were known. However, individual response rate per question differed due to participant’s willingness and ability to answer a question hence *n* varies for each variable. Data analysis was carried out using STATA 12 (Stata Corp, USA) statistical package [43]. Pearson’s chi-square (*X*^*2*^) or Fisher’s exact tests, where applicable, and t-tests were conducted to test for differences in HIV sero-status by socio-demographic factors, HIV risk factors as well as control and decision making factors. We modelled the relationship between HIV status and the three relationship power concepts (relationship control, male partner refusal of a family planning method and decision about most recent pregnancy) as focal independent variables using logistic regression. Three stepwise multivariable logistic regression models were built by entering key independent variables into the regression models. Covariates were individually added into the models. All models were adjusted for the participant’s age. In addition, we controlled for a number of potential confounders which were found to have a statistically significant association with HIV status in the bivariate analyses using *X*^*2*^ test [44, 45]. These variables included marital status, educational attainment level, age difference between woman and her partner, having ever engaged in transactional sex, ever condom use with current/most recent partner, and alcohol consumption during pregnancy. Multivariable logistic regression models were built to determine whether the key independent variable (relationship power) was related to HIV. A *p*-value of less than or equal to 0.05 (*p* ≤ 0.05) was considered as statistically significant.

## Results

Women with an unknown HIV status (*n = 91, 4.5%)* were excluded from the analysis. However, they did not statistically differ from those with a known HIV status on any of the socio-demographic characteristics, shown in Table 1. HIV prevalence among the study sample was 15.3% (CI 0.14–0.17, *n = 299/1951*).

**Table 1:** Comparison of socio-demographic characteristics among postpartum women with known and unknown HIV status

CHARACTERISTIC	HIV STATUS		*P*-VALUE
	KNOWN n (%)	UNKNOWN n (%)	
Age (years) *n = 2038*			0.29
15–19	221 (11.4)	9 (9.9)	
20–25	774 (39.8)	41 (45.1)	
26–30	534 (27.4)	21 (23.1)	
31–35	273 (14.0)	14 (15.4)	
36+	145 (7.5)	6 (6.6)	
Marital Status *n = 2041*			< 0.001
Married	1720 (88.2)	80 (87.9)	
Not Married	230 (11.79)	11 (12.0)	
Education level *n = 2037*			0.01
Primary	141 (7.3)	7 (7.7)	
Secondary	1639 (84.2)	72 (79.1)	
Tertiary	166 (8.5)	12 (13.2)	
Work Status *n = 2027*			0.75
Employed	1356 (70.0)	65 (73.0)	
Not Employed	582 (30.0)	24 (27.0)	

*A*ssociations between socio-demographic characteristics of the women by HIV status are shown in Table 2. The odds of being HIV positive increased with an increase in women’s age.

**Table 2:** Association between socio-demographic characteristics and known HIV status

CHARACTERISTIC	HIV STATUS		X^2^ TEST	UNADJUSTED OR (95% CI)	*P*-VALUE
	NEGATIVE n (%)	POSITIVE n (%)	P-VALUE		
Age (years) *n = 1947*			0.09		
15–19	196 (88.7)	25 (11.3)		ref	
20–25	667 (88.2)	107 (13.8)		1.26 (0.79–2.00)	0.33
26–30	443 (83.0)	91 (17.0)		1.61 (1.00–2.59)	0.05
31–35	225 (82.4)	48 (17.6)		1.67 (0.99–2.81)	0.05
36+	117 (80.7)	28 (19.3)		1.89 (1.04–3.37)	0.04
Marital Status *n = 1946*			< 0.001		
Not Married	174 (75.7)	56 (24.4)		ref	
Married	1477 (85.9)	243 (14.1)		0.51 (0.37–0.71)	< 0.001
Education level *n = 1946*			0.01		
Primary	108 (76.6)	33 (23.4)		ref	
Secondary	1392 (84.3)	247(15.1)		0.58 (0.38–0.87)	0.01
Tertiary	149 (89.8)	17 (10.2)		0.37 (0.20–0.70)	< 0.001
Work Status *n = 1938*			0.67		
Not Employed	1152 (84.2)	204 (15.8)		ref	
Employed	490 (85.0)	92 (15.0)		1.06 (0.81–1.39)	0.67

*UOR* Unadjusted Odds Ratio

*CI* Confidence Interval

Women aged 36 years and above were almost twice as likely to be HIV positive than the younger women aged between 15 and 19 years (UOR 1.89, 95% CI 1.04–3.37). There was a significant association between HIV status and marital status (*p < 0.001*). Marriage was protective against HIV infection; married women were two times less likely to be HIV positive compared to the unmarried women (UOR 0.51, 95% CI 0.37–0.71). The highest level of education attained had a significant association with women’s HIV status (*p = 0.01*). HIV infection decreased with increased educational attainment, women with secondary (UOR 0.58, 95% CI 0.38–0.87) or tertiary (UOR 0.37, 95% CI 0.20–0.70) education levels were less likely to be HIV positive compared to women who completed primary education only.

Table 3 shows sexual history and behavioural characteristics of the women by HIV status. Women with an age difference of ten years and more with their current/most recent partners were twice as likely to be HIV positive, than women with an age difference of less than ten years with their male partners (UOR 2.05, 95% CI 1.48–2.85). Among the women who reported forced first sex (including rape) before the age of fifteen years, more than a fifth (22.4%, *n = 67/1945*) were HIV positive. Women who had experienced forced first sex (including rape) before the age of fifteen years had higher odds of being HIV positive, compared to those who did not experience forced first sex (including rape) before the age of fifteen years (UOR 1.76, 95% CI 1.29–2.39). Our results also showed that 307 women reported ever being involved in transactional sex. Of these women, 26.7% (*n = 82/1954*) were HIV positive. Women who reported ever involved in transactional sex were 2.4 times more likely to be HIV positive than those who did not (UOR 2.40, 95% CI 1.79–3.20). Participants who reported ever being treated or diagnosed with an STI during their most recent pregnancy were nearly six times more likely to be HIV positive as compared to those who had not been treated or diagnosed of an STI during their most recent pregnancy.

**Table 3:** Association between sexual history factors, behavioural characteristics and known HIV status

CHARACTERISTIC	HIV STATUS		X^2^ TEST	UNADJUSTED OR (95% CI)	*P*-VALUE
	NEGATIVE n (%)	POSITIVE n (%)	P-VALUE		
Age difference between woman and partner *n* = 1939			< 0.001		
Less than 10 years older	1296(89.6)	204(13.6)		ref	
10 years and more older	347(74.0)	92(21.0)		2.05 (1.48–2.85)	< 0.001
Forced First sex including rape before age of 15 years *n = 1945*			< 0.001		
No	1414 (85.9)	232 (14.1)		ref	
Yes	232(77.6)	67(22.4)		1.76 (1.29–2.39)	< 0.001
Transactional sex activities with main sexual partner *n = 1954*			< 0.001		
Never	1427 (86.6)	217 (13.2)		ref	
Ever	255 (73.3)	82 (26.7)		2.40 (1.79–3.20)	< 0.001
Treated/ Diagnosed with an STI during recent pregnancy *n = 1940*			< 0.001		
Never	1577 (86.8)	240 (13.2)		ref	
Ever	65 (52.9)	58 (47.2)		5.86 (4.04–8.57)	< 0.001
Condom use with current/ most recent partner *n = 1938*			< 0.001		
Never	910 (88.5)	118 (11.5)		ref	
Ever	731 (80.3)	179 (19.7)		1.89 (1.47–2.43)	< 0.001
Alcohol use during pregnancy *n = 1947*			0.01		
No	1548 (85.3)	267 (14.7)		ref	
Yes	84 (75.8)	32 (24.2)		0.38 (0.20–0.72)	< 0.001

Table 4 shows the association between HIV status and relationship control score. Women who were HIV positive reported higher levels of control by male partner in their intimate relationships, as denoted by the higher mean score compared with HIV negative women (1.89 vs. 1.52).

**Table 4:** Association between HIV status and relationship control score

HIV STATUS	(n)	MEAN	SD	95% CI		*P*-VALUE
Negative	1584	1.52	1.33	1.45	1.58	0.01
Positive	287	1.89	1.53	1.71	2.06	

*SD* Standard Deviation

Table 5 shows the unadjusted association between decision-making factors and HIV status. Women with partners who had ever refused use of any family planning method (including condom use) were nearly 2.5 times more likely to be HIV positive compared to those whose partners had never refused use of any family planning method (UOR 2.45, 95% CI 1.61–3.73). Women who independently decided to fall pregnant for the most recent pregnancy were 0.48 times less likely to be HIV positive (UOR 0.52, 95% CI 0.29–0.95) as compared to women whose most recent pregnancy was unplanned.

**Table 5:** Association between decision-making factors and HIV status

CHARACTERISTIC	HIV STATUS		X^2^ TEST	UNADJUSTED OR (95% CI)	*P*-VALUE
	NEGATIVE n (%)	POSITIVE n (%)	*P*-VALUE		
Partner ever refused a family planning method *n = 1941*			< 0.001		
Never	1561 (85.5)	264 (14.5)		ref	
Ever	82 (70.7)	34 (29.3)		2.45 (1.61–3.73)	< 0.001
Decision to become pregnant for the most recent pregnancy *n = 1951*			0.01		
Unplanned	449 (83.5)	89 (16.5)		ref	
Woman	135 (90.6)	14 (9.4)		0.52 (0.29–0.95)	0.03
Partner	373 (80.7)	89 (19.3)		1.20 (0.87–1.67)	0.26
Both Woman and Partner	695 (86.7)	107 (13.3)		0.78 (057–1.05)	0.11

Table 6 shows three models of the association between relationship power and HIV status adjusted for covariates. When adjusted for other factors in a multivariable logistic regression model, relationship control maintained a significant association with HIV status (AOR 1.11, 95% CI 1.01–1.22). This means for every one-unit increase in relationship score among participants’ male partners, the odds of being HIV positive among the women increased by one. Having a partner who had ever refused a family planning method was significantly associated with being HIV positive (AOR 1.88, 95% CI 1.20–2.90). Making the decision about the most recent pregnancy independently was protective against HIV infection. Women who independently made the decision to fall pregnant had 0.46 less odds of being HIV positive compared to those women who had unplanned pregnancies (AOR 0.54, 95% CI 0.29–1.00).

**Table 6:** Multivariable Logistic Regression Models of the relationship between HIV status and the three focal independent variables

HIV	ADJUSTED OR (95% CI)	*P*-VALUE
Model 1: Relationship between HIV and Control in Intimate Relationships *n* = 1871, *p* < 0.001		
Relationship Control Score in Intimate Relationships		
Score	1.11 (1.01–1.22)	0.03
Model 2: Relationship between HIV and Partner Ever Refused any Family Planning Method* *n* = 1901, p < 0.001		
Current/ most recent partner ever refused a family planning method		
No	1 ref	
Yes	1.88 (1.20–2.90)	0.01
Model 3: Relationship between HIV and Most Recent Pregnancy Decision *n* = 1905, p < 0.001		
Decision about most recent pregnancy		
Unplanned	1 ref	
Woman alone	0.54 (0.29–1.00)	0.05
Both Women and Partner	1.20 (0.88–1.76)	0.20
Partner alone	0.92 (0.67–1.28)	0.64

*AOR* ***=*** *Adjusted Odds Ratio*

Models were adjusted for age; marital status; education level; age difference between woman and partner; transactional sex; *ever condom use with current/most recent partner; and alcohol consumption during current pregnancy

*Ever condom use was not adjusted for in Model 2 as it addresses contraception

## Discussion

Results show that relationship power was associated with HIV infection among the postpartum women. This relationship was demonstrated in three ways, of the gender power factors used in this study. Firstly, the relationship control score indicating male dominance in intimate relationships was related to the risk of HIV infection among the women. Secondly, a male partner’s ever refusal to use any family planning method was linked to the women’s HIV positive status. Lastly, a woman making an independent decision to have fall pregnant in their most recent pregnancy was protective against HIV positivity.

The study tested the hypothesis that a high level of control by male partners was associated with HIV positivity among low-income postpartum women attending public PHC clinics in Zimbabwe. It is in low-income areas that people without medical insurance utilize public government funded clinics. In high or middle-income areas, there are fewer clinics, and most users have medical aid and do not attend these. We expected that the relationship between relationship-control and HIV among postpartum women attending low-income public clinics be different from other contexts. Violence has been reported to be high in low-income settings. It is a factor of poverty and deprivation, manifests where resources are limited, it fields violence and is fueled by violence [40, 46].

Lack of power in heterosexual relationships has been found to increase vulnerability to HIV positivity among women [12, 27, 47, 48]. Our study found an association between women whose current or most recent partners had ever refused use of a family planning method and HIV positivity. Results showed that women whose current or most recent partners had ever refused use of any family planning method were more likely to be HIV positive, than women whose partners had never refused any family planning methods. This finding suggested male dominance in reproductive decision-making and its impact thereof on HIV vulnerability among women in intimate relationships. Men’s social power is also evident in their verbal opposition to family planning [49]. A male partner’s refusal of family planning use implies a desire to make a partner pregnant without her consent, which is linked to or is achieved through verbal threats, unprotected forced sex, and or contraceptive sabotage [50], which violates the women’s sexual and reproductive health and rights. Such actions may result in unwanted pregnancies and increased vulnerability to HIV infection.

Women who made independent decisions about falling pregnant were less likely to be HIV positive. This shows that the agency of making independent decisions may be a sign of power in an intimate relationship. Women who made the decision to become pregnant must have been more assertive and better able to make safer sex decisions in their lives or partnerships. Thus, our study further demonstrated that women who had an increased autonomy were empowered to make better decisions about their health. Since almost all HIV transmission cases to women (97%) occurs through sexual intercourse [51], women who exercise adequate relationship power on sexual behaviour and choices are more able to protect themselves against HIV positivity. However, the role of multi-sexual concurrent sexual partnerships have also been recognized as important for the transmission of STIs especially among heterosexual HIV transmission in Africa [52].

In the Zimbabwean context, it has been noted with concern that even if a couple may discuss family issues, in most relationships the husband still has the final say [10]. This includes decisions about the size of the family [10, 14]. Traditional family norms are based on the supremacy of the male members of the household, and the subordination and dependency of women [10, 53]. When measuring decision-making in the context of this study we looked at the outcome of decision-making. However, decision making is a dynamic process which may be characterised by compromise [54]. The fact that pregnancy decision-making by mutual partners was not found to be protective against HIV positivity in this study could be attributed to one of the partners compromising to please the other. This may be an indication of limited agency if the woman was the one who compromised. Women may be more likely to compromise as gender roles and norms may influence their desire to please their partner [24, 55, 56]. In this regard, women may knowingly, or unknowingly complicit in perpetuating gender roles and norms. Further research is needed to evaluate if decision making involving women, whether independently or jointly with their male partners as compared to when women are not involved heightens the risk of HIV positivity.

Our findings that male dominance in intimate relationships increases women’s vulnerability to HIV positivity are similar to several previous studies conducted in South Africa and other parts. A cross-sectional study among low income women attending ANC in South Africa found that women who reported a high score on the SRPS, which signified male dominance in the intimate relationship had greater odds of being infected with HIV [47]. In rural Eastern Cape Province, another study found that ANC women, whose current or most recent relationship scored high on the SRPS were more likely to be infected with HIV [57]. A cohort study in rural South Africa among women who were HIV negative at the beginning of the survey showed that more women with low relationship power became HIV infected as compared to 73 of the 704 women with medium or high relationship power [48].

In this study, we used a surrogate variable to measure control in intimate relationships. Previous studies on relationship power have used a validated SRPS scale [12, 42, 58, 59], which we did not use in this analysis. The 23-item SRPS scale contains conceptual dimensions of relationship power dynamics [26], to evaluate the association between relationship power and HIV, and significant associations have been reported [60]. In an earlier study, Pettifor (2004) found, control in intimate relationships to decrease the probability of consistent condom use, and this in turn increased the risk of HIV positivity among women aged 15–24 years [12]. This association between the SRPS and consistent condom use in intimate relationships, highlights relationship power as an important aspect of safe sex decision-making [42]. Other studies have also found women with high relationship power levels to have a higher likelihood of reporting consistent condom use in their relationships [12, 42, 58, 59]. We acknowledge that the relationship between control in intimate relationships and HIV sero-positive status among our study population might be more complex than we assumed, for example, an HIV positive status might have triggered more controlling behaviour from male counterparts in this study.

Gender power imbalances that manifest in men being the sex initiators or the ones’ who set the conditions for sexual encounters [10], result in relationship power imbalances which limit the ability of women to protect themselves against HIV infection from their sexual partners [8]. This is because they are unable to negotiate for safe sex, even if they are aware that they are being exposed to HIV. Although gender inequality has been recognised as an important contextual factor in HIV risk, prevention programmes have not resulted in greater gender equality. Gendered power continues to influence HIV risk in at least heterosexual transmission of HIV.

Results of this paper have to be interpreted in light of various limitations. The study was cross-sectional, which makes it impossible to ascertain causality between control or decision-making in intimate relationships and HIV sero-status among the women under study. This analysis focused on postpartum women whose HIV status was known, excluding those with an unknown status (4.5%). Women with known HIV status did not differ from women with unknown status, in terms of demographic characteristics. However, women with unknown HIV status may have been different in terms of other variables that were considered in the logistic regression models. Ideally, relationship power is measured using the SRPS, which contains Relationship Control and Decision Making sub-scales. The SRPS was not used in the primary study.

## Conclusions

Our study showed that lack of power among women in sexual relationships is related to women’s increased vulnerability to HIV positivity. This provides further evidence that male dominance in intimate relationships increases women’s vulnerability to HIV. Gendered power continues to influence HIV risk in at least heterosexual transmission of HIV. This suggests that prevention efforts have not successfully resulted in gender equality. Findings from this paper highlight the public health importance of understanding the consequences of relationship power imbalances in preventing risk of HIV infection among women, particularly pregnant and post-partum women. Effective HIV prevention interventions should target gender power differences between men and women to try and improve women empowerment [61]. Social and behavioural change communication (SBCC) interventions should be designed to target such gendered health related problems nested in HIV control initiatives. However, we still do not know the impact of policies to change relationship control.

## Data Availability

The datasets used and/or analysed during the current study are available from the corresponding author on reasonable request.

## Abbreviations

AIDS: Acquired Immune Deficiency Syndrome
ANC: Ante-natal Care
AOR: Adjusted Odds Ratio
CI: Confidence Interval
GBV: Gender Based Violence
HIV: Human Immunodeficiency Virus
IPV: Intimate Partner Violence
PHC: Primary Health Care
PNC: Post-natal Care
SBCC: Social and Behaviour Change Communication
SRPS: Sexual Relationship Power Scale
STI: Sexually Transmitted Infection
UOR: Unadjusted Odds Ratio
WHO: World Health Organization
